# Postoperative Infections After Appendectomy for Acute Appendicitis: The Surgeon’s Checklist

**DOI:** 10.3390/antibiotics14090954

**Published:** 2025-09-20

**Authors:** Martina Leandri, Carlo Vallicelli, Giorgia Santandrea, Daniele Perrina, Francesca Bravi, Massimo Sartelli, Federico Coccolini, Luca Ansaloni, Vanni Agnoletti, Fausto Catena

**Affiliations:** 1 Department of General and Pancreatic Surgery, The Pancreas Institute, University of Verona Hospital Trust, 37134 Verona, Italy; martina.leandri@studenti.univr.it; 2 General, Emergency and Trauma Surgery Department, Maurizio Bufalini Hospital, 47521 Cesena, Italy; 3 Healthcare Administration, Santa Maria Delle Croci Hospital, 48121 Ravenna, Italy; 4 Department of Surgery, Macerata Hospital, 62100 Macerata, Italy; massimo.sartelli@sanita.marche.it; 5 Department of General, Emergency and Trauma Surgery, Pisa University Hospital, 56124 Pisa, Italy; 6 Department of Surgery, Fondazione IRCCS Policlinico San Matteo, University of Pavia, 27100 Pavia, Italy; 7 Anesthesiology and Intensive Care Unit, Maurizio Bufalini Hospital, 47521 Cesena, Italy; 8 Department of Medical and Surgical Sciences, University of Bologna, 40126 Bologna, Italy

**Keywords:** appedectomy, surgical site infection, intra-abdominal abscesses, abdominal drain, stump closure, peritoneal irrigation, prevention, emergency surgery

## Abstract

Acute appendicitis remains one of the most common surgical emergencies, with a lifetime incidence of approximately 7–8% in the USA and Europe. Despite the widespread adoption of the laparoscopic approach and advances made in perioperative care, post-operative infections—particularly intra-abdominal abscesses—continue to pose a substantial clinical challenge, with an overall probability that ranges from 5 to 15%. Nowadays, it is essential not only to improve patient outcomes by reducing these complications but also to promote responsible antibiotic use. This review provides an in-depth examination of post-appendectomy infections in adults, synthesizing research from the past decade. It explores the various risks involved, including those related to the patient, the disease itself, and the surgical techniques employed. There is particular emphasis on the impact of surgical approach, closure methods, timing of surgery, and intraoperative decisions such as drain placement, peritoneal lavage, and routine bacterial cultures. Part of the discussion is about emerging data regarding the use of antiseptic solutions and specimen retrieval techniques. Additionally, the review examines current approaches to managing postoperative intra-abdominal abscesses. It assesses when antibiotics are necessary, evaluates image-guided percutaneous drainage, and considers laparoscopic re-intervention as a possible solution. While recent studies offer valuable insights, the heterogeneity of available evidence highlights the pressing need for high-quality, standardized research. Ultimately, a deeper understanding of infection pathways and preventative strategies is vital—not only for reducing morbidity and hospital readmissions, but also for safeguarding the long-term efficacy of antibiotics and delivering safer, more effective surgical care.

## 1. Introduction

Acute appendicitis remains one of the most common surgical emergencies worldwide, with a lifetime incidence in the USA and Europe around 7–8% [1], with appendectomy representing the standard treatment in the vast majority of cases. Post-operative infections—including superficial and deep surgical site infections (SSI), intra-abdominal abscesses (IAA), and, in severe cases, sepsis—continue to pose a relevant challenge, particularly in complicated forms of the disease. The incidence of post-operative infection after appendectomy is highly variable in the literature, depending on the population under investigation, the surgical approach, and whether there was a consistent definition of infection employed. The average SSI incidence reported is between 5% and 15%, and the incidence is higher in complicated appendicitis. An extensive retrospective study conducted in China on over 9000 patients found a cumulative SSI rate of 6.2%, with 3.7% attributed to incisional infections and 3.0% to organ/space infections, including intra-abdominal abscesses. Notably, the transition from open to laparoscopic appendectomy (LA) was associated with a significant reduction in incisional SSI rates, from 6.7% to 2.2% over the study period [2]. Similarly, a European multicenter analysis reported an overall SSI rate of 6.6%, with organ/space infections accounting for nearly 4.6%—a majority of which were associated with complicated appendicitis [3]. More recently, a 2023 meta-analysis including over 5000 patients confirmed that the risk of post-operative infection in complicated appendicitis—defined by gangrene, perforation, or abscess—was substantially higher than in uncomplicated cases, with infection rates approaching 15.4% [4].

This narrative review provides an up-to-date overview of the literature regarding risk factors for post-operative infection following appendectomy for acute appendicitis and discusses possible preventive strategies. Emphasis is placed on both patient- and disease-related variables, surgical techniques, and perioperative practices, to offer practical insights supported by the most recent evidence published between 2015 and 2025.

## 2. Materials and Methods

We searched through the Medline National Library of Medicine and Cochrane Central Register of Controlled Trials (CENTRAL) using the following terms: “peritoneal infection appendectomy adult”, “time and outcomes appendectomy”, “intra-abdominal infections appendectomy”, “peritoneal lavage appendectomy”. MeSH terms were included. We also conducted a hand-search of references for previous reviews on the same topic to include all relevant studies. We included studies published between January 2015 and 2025, excluding case reports. We defined postoperative infections according to the criteria outlined by the Centers for Disease Control and Prevention (CDC), as described in the January 2025 edition of the National Healthcare Safety Network (NHSN) Patient Safety Component Manual [5].

## 3. Post-Operative Infections Risk Factors

### 3.1. Patient-Related Factors

Various risk factors for post-appendectomy infection have been outlined in the recent literature to reflect the multifactorial etiology of this complication. In patient-related factors, advanced age has always been associated with risk, particularly among patients over the age of 60 years, who are at greater susceptibility to sepsis and wound infections. While body mass index (BMI) is often used as a general marker, visceral adipose tissue (VAT) has shown more substantial predictive value for surgical site infections (SSIs). A 2023 retrospective multicenter study involving 441 patients found that visceral obesity, assessed via CT scan, was significantly associated with an increased risk of incisional infections (OR 2.68; 95% CI 1.16–5.85) [3,6].

### 3.2. Disease-Related Factors

From a disease-related perspective, the intrinsic characteristics of the appendicitis itself—namely, severity, intraoperative findings, and timing of intervention—are among the strongest predictors of post-appendectomy infectious complications. Complicated appendicitis (perforated, gangrenous, or associated with abscess/phlegmon) remains the single most significant disease-related risk factor. A comprehensive meta-analysis combining data from 35 studies, including over 5300 patients, reported a postoperative infection rate of 15.4% in cases with complications [4]. Multivariate analyses in large cohort studies consistently show that complicated appendicitis increases the odds of surgical site infection by approximately fourfold compared to uncomplicated cases [6]. This heightened risk stems from greater bacterial load and tissue damage inherent to advanced disease. 

The extent of intra-abdominal contamination during surgery—particularly the presence of purulent or feculent peritoneal fluid—is a direct indicator of the risk of organ/space infection. The WSES Jerusalem Guidelines (2020) recommend aspirating rather than leaving contaminated fluid to settle, as contamination consistently correlates with abscess formation [QoE: Moderate; Strength of recommendation: Strong; 1B] [7]. Large observational series confirm that intraoperative “dirty” fields are associated with a higher incidence of abscesses and require vigilant postoperative management [6].

The ideal timing of surgical intervention in acute appendicitis remains problematic, namely, its impact on the risk of infectious postoperative complications. While traditional dogma has encouraged early appendectomy, recent publications have moderated this dogma. Brief controlled delays have been suggested to be safe in well-selected patients. A growing body of evidence indicates that in-hospital delays of up to 24 h from admission do not significantly increase the risk of surgical site infections, particularly in patients with uncomplicated appendicitis.

A 2024 meta-analysis including over 500,000 patients demonstrated that while delays beyond 24 hours were associated with a higher incidence of complicated appendicitis and overall morbidity, they were not independently associated with increased rates of postoperative infection [8]. These findings suggest that the evolution of the disease process before hospital admission—rather than the in-hospital delay itself—may play a more decisive role in postoperative outcomes. This view is supported by a network meta-analysis of 232,678 patients, which demonstrated that appendectomy within 24 h is associated with better outcomes (including lower rates of complicated disease, shorter length of stay, and reduced mortality), but not necessarily a lower rate of SSI when compared with surgeries performed later [9]. In other words, while early surgery improves overall recovery, it does not definitively reduce postoperative infection rates if performed within 24 h, as reported in Table 1.

- For uncomplicated appendicitis in a stable patient, an appendectomy within 24 h of presentation is a reasonable clinical practice without undue risk for surgical-site infection.
- For patients with suspected complicated appendicitis—characterized by an acute presentation with severe abdominal signs, elevated markers of inflammation, and/or appropriate imaging—urgent surgery should take place as soon as possible—ideally within 8 to 12 h of arrival to minimize the extent of disease progression and complications.
- Ultimately, a timely presentation and diagnosis are paramount because total disease duration has more bearing on outcomes than the duration of in-hospital observation.

### 3.3. Surgical Factors

Several surgical factors also influence the rates of infection. The nature of the surgical procedure employed is also a critical factor: open appendectomy has always been associated with higher rates of SSI and sepsis incidence compared to laparoscopic procedures. A multicenter study documented twice the risk of sepsis following open surgery, further supporting the benefit of minimal access procedures [10]. This issue was the study by Peng et al. (2018), which assessed the influence of pneumoperitoneum on systemic inflammation and bacterial translocation in adults with peritonitis caused by acute appendicitis. The authors found that laparoscopic approaches using CO_2_ pneumoperitoneum (CDP) were associated with reduced systemic inflammatory response (lower IL-6, CRP, and TNF-α levels) compared to open surgery, indicating that the performance of laparoscopy operation under CDP is feasible to control SIRS [11]. 

Among the modifiable risk factors for post-appendectomy infections, the technique used to close the appendix stump during LA has garnered significant attention.

Historically, endoloops—preformed absorbable sutures—were the most common choice. However, emerging evidence has highlighted the advantages of non-absorbable polymeric Hem-o-lok (H-O-L) clips, especially in reducing intra-abdominal abscesses and streamlining operative efficiency. A retrospective study by Soll et al. (2016), which evaluated 813 consecutive LAs, demonstrated that closure with Hem-o-lok clips was associated with a significantly lower rate of intra-abdominal abscesses compared to endoloops [12]. While a 2020 study involving 673 patients across uncomplicated and complicated appendicitis cases reported similar abscess rates for both techniques when data were stratified, the rates consistently favored Hem-o-lok. In uncomplicated cases, abscess occurrence was 1% vs. 3%, and in complicated cases, 2% vs. 6% for endostapler, with no significant difference in clip vs. stapler comparison. [13]. These findings indicate that Hem-o-lok clips perform at least as well as staplers, while being cost-effective and simpler to use.

A 2023 observational study from Doha [14] corroborated these findings in a smaller cohort (123 patients), showing no difference in postoperative complications or readmissions between Hem-o-lok and endoloop techniques. Despite its limited size, this study reinforces the safety profile of Hem-o-lok usage. Multiple studies have emphasized the operational benefits of Hem-o-lok clips. A Polish randomized trial noted significantly shorter operative times with Hem-o-lok (approximately 38 min) compared to endoloop or stapler, without compromising infection rates. Conversely, endoloops were associated with longer operative durations, likely due to the need for knot tying and securing the ligature [15].

Moreover, a material cost analysis reveals substantial savings with clips: a kit of 5 Hem-o-lok clips costs approximately EUR 25, compared to around EUR 360 for an endostapler. This dramatic difference makes Hem-o-lok the more economical option without sacrificing safety.

The clip application is technically straightforward, but not without constraints. Hem-o-lok clips can safely secure an appendix base up to approximately 10 mm in diameter; beyond this threshold or in the presence of severe base inflammation, endoloops—or staplers—may still be preferred [16]. Furthermore, because Hem-o-loks are non-absorbable, there is a small theoretical risk of clip migration; however, this has not translated into clinically significant downstream complications in the reviewed literature. The most recent WSES Jerusalem Guidelines (2020) concluded that there is no definitive advantage for staplers over endoloops in routine cases; however, they highlighted that polymeric clips, including Hem-o-lok, offer a cheaper and easier alternative in appropriately selected patients. [QoE: Moderate; Strength of recommendation: Strong; 1B] [7].

Overall, the risk of post-operative infection following appendectomy is determined by a complex interplay of host, disease, and procedural factors. Recognizing and mitigating these risks is crucial for enhancing surgical outcomes and minimizing the impact of post-appendectomy complications.

Major studies investigating the appendix stump closure technique are reported in Table 2.

## 4. Preventions and Possible Solutions

Despite advances in surgical technique and perioperative care, postoperative infection remains one of the most frequent complications of appendectomy for acute appendicitis. More recently, interest has shifted from the simple identification of risk factors to the actual alteration of intraoperative and perioperative practices in an attempt to minimize infectious morbidity. Several procedural techniques have been proposed, including the use of abdominal drains, intraoperative cultures, and peritoneal irrigation techniques. Yet the evidence for each such intervention remains heterogeneous, and clinical guidelines are not always willing to make firm recommendations.

### 4.1. Abdominal Drainage: Protective or Redundant?

It is presumed to have the ability to evacuate residual infectious material and minimize the formation of abscesses. However, recent high-quality evidence challenges this long-standing belief. A pivotal 2025 Cochrane review, which included eight randomized controlled trials and quasi-randomized studies encompassing 739 children and adults with complicated appendicitis, concluded that drainage did not meaningfully reduce intra-abdominal abscesses, wound infection rates, or overall complications. Alarmingly, this comprehensive analysis suggests that drains may increase mortality and lengthen hospital stay: analyzing perforated and gangrenous appendicitis cases confirmed that drainage did not provide a protective effect against postoperative intra-abdominal infections, while being linked to a higher incidence of superficial wound infections, fistula, bowel obstruction, ileus, and length of hospital stay [17]. International guidelines [7], have progressively moved away from endorsing routine intra-abdominal drainage in appendicitis, especially in the context of minimally invasive surgery [QoE: Low; Strength of recommendation: Weak; 2C]. These positions reflect a consensus toward individualized, selective use of drains, rather than prophylactic placement. As laparoscopic techniques and intraoperative suction-irrigation tools have improved, drainage has become increasingly obsolete in the majority of appendectomy cases.

### 4.2. Intraoperative Culture Swabs (ICS): Necessary Investigations or Waste of Resources?

Surgeons have traditionally collected peritoneal cultures during adult appendectomy—particularly in perforated or gangrenous appendicitis—believing that identifying pathogens could inform targeted postoperative antibiotic therapy. Yet, emerging literature in the past decade has cast doubt on whether this practice benefits patients.

A 2019 prospective cohort study by Akingboye et al. assessed 141 adults with complicated (perforated or gangrenous) appendicitis at a UK center. Although 25% of cases yielded positive cultures, only nine results were available before discharge, and none prompted a change in antibiotic therapy. Even when resistance was identified, a broad-spectrum switch was made instead of a targeted adjustment. The authors concluded that routine pus sampling “remains of little clinical value” for adults undergoing appendectomy [18]. Similarly, Peña et al. examined this issue directly. In their single-center cohort study in Argentina, data were collected from all patients over 16 years old who underwent LA for complicated appendicitis from January 2008 to January 2018. The study included patients with gangrenous appendicitis (confirmed by histopathological report) and peritonitis (confirmed intraoperatively). A total of 1639 LAS were performed. Of these, 270 (16.5%) were complicated appendicitis; 90 received ICS. Interestingly, although the antibiotic regimen was adjusted based on culture findings in all cases, this practice did not appear to reduce postoperative morbidity, as patients with an already adjusted antibiotic treatment were significantly more likely to develop postoperative IAA (21% vs. 9.4%). A potential explanation of these findings could be that patients with culture swabs had more severe disease than those without. ICS and antibiotic adjustment to the initial pathogen were not associated with a lower incidence of postoperative IAA and did not modify the treatment needed for this complication [19].

In contrast to more conservative views, a Colombian retrospective cohort (2023) focusing on 144 adults with complicated appendicitis has shown a more proactive outcome. Over 56% of patients who underwent intraoperative aerobic cultures had their antibiotic regimens adjusted based on culture results. A subsequent reduction in surgical site infections was reported, with cultures showing a significant decrease in the risk of SSI [20]. 

While promising, these findings are based on a single-center experience, and broader validation is needed to confirm their generalizability. Anyway, international guidelines now reflect these divergent findings. The WSES guidelines explicitly advise against routine use of intraoperative cultures for adult appendicitis and recommend their application in the case of immunocompromised patients, nosocomial infections, or in cases with known exposure to resistant organisms [Recommendation 1C] [7].

### 4.3. Peritoneal Irrigation and Appendix Extraction: Efficacy, Techniques, and Evolving Evidence

The employment of peritoneal lavage during appendectomy, particularly in complicated or perforated acute appendicitis, is an issue of considerable controversy. Abdominal irrigation has been used routinely in the past as a means of reducing bacterial loads and avoiding postoperative IAAs or SSIs. Good-quality trials challenged the validity of this argument in recent years.

The randomized controlled trial by Gemici et al. compared peritoneal lavage (500 mL saline) versus aspiration alone in 286 adult patients with perforated appendicitis undergoing LA. Their findings revealed no significant difference in the rates of postoperative intra-abdominal abscesses, wound infections, or hospital stays between the two groups. The study concluded that lavage provided no additional benefit and might be unnecessary in the routine management of complicated appendicitis during laparoscopy [21]. A comprehensive 2021 meta-analysis in the World Journal of Emergency Surgery, including 5315 patients across 17 studies, agreed with Gemici et al., concluding that suction alone was at least equivalent to irrigation in preventing abscesses and significantly better in terms of operative time and reoperation rates [22].

However, further nuance on this topic was introduced by Wang et al. (2021) in a retrospective cohort study: the patients were divided into two groups based on the sequential order in which peritoneal irrigation, suction, and extraction of the appendix had been performed. In Group 1, peritoneal irrigation and suction had been performed before extraction of the appendix, and in Group 2, they had been performed after extraction of the appendix. The final study sample included 571 patients, 116 (20.3%) in Group 1 and 455 (79.7%) in Group 2. The incidence of surgical wound infection was lower in Group 1 (6.9%) than in Group 2 (14.1%) (*p* = 0.038). There were no significant differences in the rates of intra-abdominal abscess, small bowel obstruction, or readmission within 30 days between the two groups (*p* > 0.05) [23].

Recent studies explore the potential of antiseptic or antimicrobial solutions, such as super-oxidized agents, which may redefine best practices in managing intra-abdominal contamination. In 2024, a randomized clinical trial from the Department of Surgery at the Queen Elizabeth Hospital, Malaysia, the PLaSSo trial, analyzed the effect of peritoneal and wound lavage with super-oxidized solution in open appendicectomy for perforated appendicitis. The findings from the study suggest a statistically significant reduction in SSIs (both deep and superficial) in the intervention group compared to the saline lavage group, without an increase in operative time or adverse events. This supports the hypothesis that antimicrobial properties of irrigating solutions may be more important than the mechanical act of lavage itself [24].

Although adult data on antiseptic lavage in laparoscopic procedures are not yet available, a 2016 RCT in general peritonitis (not appendicitis-specific) showed that super-oxidized solution significantly reduced bacterial load and shortened hospital stay compared to saline [25]. These findings suggest that antiseptic solutions offer measurable benefits when adapted for use in laparoscopic procedures. A summary of the most recent studies described above is presented in Table 3.

As much as peritoneal lavage, the method of extracting the appendix from the abdominal cavity is also of interest in the field of postoperative infections and is an essential step within the surgical procedure itself. Traditionally, the use of a specimen retrieval bag (endobag) has been advocated to prevent contamination of the port site and subsequent surgical site infections (SSIs).

However, recent evidence suggests that this precaution may not provide a significant clinical advantage. In an extensive retrospective cohort study, Turner et al. analyzed outcomes in 4646 adult patients undergoing LA for both uncomplicated and complicated appendicitis. The study found no significant difference in the incidence of postoperative SSIs between those who had the appendix removed with a specimen retrieval bag and those who did not [26]. It appears clear how, in the era we are living in, where healthcare costs are a significant focus of political debate, other protected techniques of specimen retrieval, described in the literature, could be employed to decrease overall costs, such as using the cut end of a glove finger [27].

According to a selective approach, recommending bag use primarily when:

1. Gross contamination is present (e.g., gangrene, spillage);
2. The appendix is being removed through an enlarged port site;
3. Institutional infection control data suggest benefit.

In contrast, simple direct extraction via a trocar port may be considered both safe and efficient in the majority of uncomplicated and carefully managed laparoscopic cases, aligning with principles of cost-effectiveness and minimally invasive practice.

## 5. Post-Appendectomy Intra-Abdominal Abscesses

Persistent intra-abdominal sepsis and development of intra-abdominal abscess (IAA) is a significant complication following both laparoscopic and open appendicectomy. The overall rate of postoperative IAA formation is low, ranging from 2 to 3% [2]. Considering the large number of appendectomies performed, this complication affects a significant number of people in our communities.

### 5.1. Medical Management with Antibiotic Therapy

Once an intra-abdominal abscess is radiologically confirmed following an appendectomy, antibiotic therapy represents a cornerstone of conservative management, particularly in clinically stable adult patients and in cases where the abscess is small, well-localized, and not associated with signs of sepsis or generalized peritonitis. This approach has gained increasing support as a safe alternative to percutaneous or surgical drainage, provided that careful patient selection and close clinical monitoring are employed.

Initial empiric antibiotic regimens should provide broad-spectrum coverage, including both Gram-negative aerobes and anaerobes, which are the most frequently isolated pathogens in post-appendectomy infections.

According to Sartelli et al.’s review, standard empirical options include piperacillin–tazobactam, ceftriaxone combined with metronidazole, or a carbapenem such as ertapenem in settings with high prevalence of resistant organisms. For patients with severe β-lactam allergy, a combination of fluoroquinolones and metronidazole may be considered [28].

The choice of antibiotics should ideally be refined based on microbiological data, mainly when intraoperative cultures or image-guided drainage samples have been obtained.

The role of targeted therapy is crucial in minimizing the risk of treatment failure, avoiding overtreatment, and reducing the selection of resistant organisms. In this context, Cho et al. (2016) have conducted a prospective cohort study of over 1800 patients undergoing LA, showing that the use of routine postoperative antibiotics did not avoid abscess development but confirmed the need for individualized treatment decisions according to clinical presentation and imaging findings rather than prophylactic or empirical alone [29].

Once antibiotic therapy has been initiated, its duration should be guided by clinical response and adequacy of source control. Evidence from the STOP-IT randomized trial demonstrated that a 4-day antibiotic course was non-inferior to more prolonged therapy (up to source resolution) for composite outcomes including SSI, recurrent intra-abdominal infection, and mortality [30].

Switch to oral therapy is appropriate once the patient is afebrile, tolerates oral intake, and shows clinical improvement. Rescan by radiology after 5 to 7 days of treatment is often required to prove resolution or to guide further intervention if the abscess does not diminish or grow. While antibiotics alone can be curative in selected patients, failure to respond within the first few days of treatment should prompt reconsideration of source control measures, including image-guided drainage. In general, the surgical management of post-appendectomy abscess, when appropriately indicated, offers a non-surgical and efficacious solution. However, it depends on early identification, appropriate patient selection, and strict follow-up, along with strict adherence to evidence-based principles of antimicrobial stewardship [28].

### 5.2. Image-Guided Percutaneous Drainage

While percutaneous drainage, guided by US and CT scans, is widely adopted for managing intra-abdominal abscesses following complicated appendicitis, specific evidence regarding its role in post-appendectomy abscesses, particularly in adult patients, is limited. Most available studies focus on abscesses developing in the setting of non-operative management or during initial perforated appendicitis. A French case series involving patients with postoperative abdominopelvic abscesses- after general surgery—reported a 78% success rate, with residual fluid after the initial procedure being the most significant predictor of failure [31].

**Figure 1:**
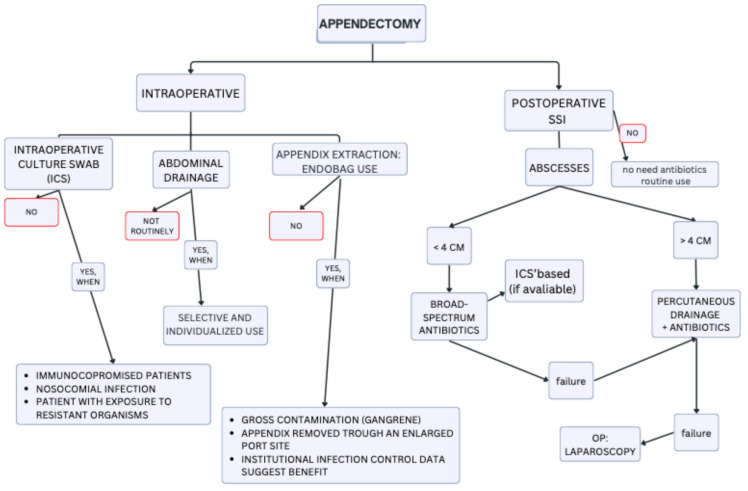
Post-Appendectomy Infections: Stepwise Management Flowchart.

These data suggest that percutaneous drainage may be adequate in well-demarcated abscesses ≥4 cm; conversely, smaller abscesses (< 4 cm) can often be managed conservatively with antibiotics alone, provided the patient remains clinically stable. A flowchart summarizing the possible management strategies is presented in Figure 1. However, current guidelines give only general recommendations, often extrapolated from studies involving peri-appendiceal or intra-abdominal abscesses in broader surgical contexts.

An apparent lack of high-quality, prospective data remains regarding post-appendectomy abscesses, specifically regarding optimal timing, patient selection criteria, and long-term outcomes of percutaneous drainage versus conservative management or reintervention. In this context, future research must create definitive indications for percutaneous drainage postoperatively, ideally grounded on multicenter prospective studies or randomized controlled trials. Developing robust clinical, radiological, and microbiological predictors of success or failure would significantly improve patient stratification and guide individualized treatment decisions.

### 5.3. Re-Intervention: Is Laparoscopy Safe?

In cases where conservative management fails—due to multiloculated abscesses, persistent sepsis, or failed drainage—surgical reintervention becomes necessary. Traditionally performed via laparotomy, recent studies support re-laparoscopy as a safe and effective alternative in selected patients. The survey by Mahmood et al. (2019), titled “Early Laparoscopic Washout May Resolve Persistent Intra-Abdominal Infection Post-appendicectomy”, demonstrated that timely laparoscopic washout in adult patients with persistent or recurrent intra-abdominal infection resulted in favorable outcomes, with low morbidity and rapid resolution of sepsis. This retrospective review emphasized that early re-laparoscopy—when performed in hemodynamically stable patients—can offer a safe and effective alternative to open surgery or prolonged percutaneous drainage [32]. Similarly, Casas et al. (2021), drawing on a single-center experience spanning 14 years, investigated whether it is feasible and practical to maintain a minimally invasive approach even in the context of complex post-surgical scenarios. It was concluded that re-laparoscopy for complications following initial LA is feasible in over 80% of adult patients, maintaining the benefits of minimally invasive surgery even in the setting of postoperative sepsis or abscesses [33]. 

Despite these encouraging outcomes, some limitations and contraindications must be appreciated. Laparoscopic reoperation requires adequate skill and access to surgical facilities, which perhaps may not be available everywhere. The hemodynamically unstable patients, who have diffused peritonitis or have bulky intra-abdominal adhesions, cannot be candidates for laparoscopy and would be best treated with open surgery. The presence of multiple poorly localized abscesses can lower the effectiveness of laparoscopic washout and pose difficulty in visualizing. Furthermore, evidence favoring this strategy remains limited to small, retrospective series, often from high-case-volume institutions with specialized expertise. No standard exists as yet on the optimal timing, patient selection criteria, and surgical techniques to be employed. Additional large-scale prospective studies are required to further define the role of laparoscopy in this context.

The application of ERAS to elective abdominal surgery has been widely successful, significantly reducing hospital stay, complications, and improving patient experience. But how does ERAS perform in the emergency setting of appendectomy, where the primary goal is often managing infection rather than electively optimizing recovery? ERAS appendectomy trials have not consistently demonstrated statistically lower SSI rates, but larger meta-analyses across gastrointestinal surgery report promising trends. A prospective, randomized controlled trial in Poland evaluated a modified ERAS protocol in adult patients undergoing LA. Elements included patient education, avoidance of drains, local anesthesia, low-pressure pneumoperitoneum, early mobilization, and prompt resumption of diet. This protocol reduced the length of stay (median 1.25 vs. 2.0 days), significantly lowered early pain scores, and resulted in 42% of patients being discharged within 24 h—without increasing readmission or infection rates [34]. Similarly, a Chinese RCT from 2024 with 120 adults found that ERAS implementation accelerated bowel function recovery, decreased hospitalization time and costs, and reduced pain, all without affecting postoperative complication rates—including SSI [35]

A comprehensive 2024 meta-analysis of ERAS across gastrointestinal surgeries, including appendectomy subsets, revealed a trend toward lower SSI (OR 0.80; 95% CI 0.39–1.64), although this did not reach statistical significance. However, ERAS notably reduced the incidence of pulmonary infections (OR 0.44) [36]. 

We can conclude that while direct SSI reduction has not been conclusively proven in appendectomy-specific RCTs, trends are favorable, and broader GI surgery data suggest moderate SSI risk reduction.

## 6. Limitations

While this narrative review amply summarizes the current literature regarding post-operative infections following appendectomy for acute appendicitis, it needs to be appreciated that it has its limitations. Narrative reviews, unlike systematic reviews or meta-analyses, lack a rigorous methodological approach towards study selection, data extraction, and quality assessment. Therefore, there is no greater risk of selection bias since the choice of studies is based on personal opinion and could not possibly represent all the evidence. Furthermore, differences in study design, patient population, definition of infection, and operation technique throughout the included papers could diminish the applicability of findings and complicate direct comparisons.

Additionally, the heterogeneity in outcome measures and reporting styles across studies hinders the robustness of quantitative synthesis. Some relevant data may have been inadvertently overlooked despite efforts to include studies published between 2015 and 2025, and only in adult populations. Finally, while recent and high-quality publications were prioritized, the lack of formal quality appraisal tools may allow for the inclusion of studies with potential methodological weaknesses.

Nevertheless, narrative reviews such as this one remain valuable in highlighting emerging trends, identifying areas of consensus and controversy, and guiding future research directions—particularly in rapidly evolving clinical domains like minimally invasive surgery and infection prevention strategies.

## 7. Discussions

Postoperative infection following appendectomy for acute appendicitis remains a significant clinical issue, especially with complicated diseases. Even with enhanced surgical techniques and perioperative management, intra-abdominal abscess (IAA), surgical site infection (SSI), and systemic complications remain a hindrance to patient recovery and resource utilization.

Risk of infection is multifactorial, being reliant on patient-related factors such as age, obesity, diabetes, and immunosuppression, and disease-related factors such as severity of appendicitis, perforation, peritonitis, and delay to operation. Notably, growing evidence suggests that delays exceeding 24–48 h in complicated appendicitis can exacerbate infectious outcomes, though immediate surgery may not always be necessary in uncomplicated cases.

Surgical technique also plays a crucial role in preventing infections. LA has consistently shown lower SSI rates compared to open surgery, particularly in uncomplicated cases. The method of stump closure—whether by endoloop or Hem-o-lok—may influence the risk of intra-abdominal infection, though current data remain inconclusive. Additionally, adjuncts such as the use of specimen retrieval bags or intraoperative cultures have not demonstrated a consistent benefit in adult populations, and their routine use should be carefully evaluated in light of cost and outcome data.

Controversies persist regarding the utility of peritoneal drainage and peritoneal lavage. Recent literature, including Cochrane reviews and randomized trials, has increasingly questioned the routine use of drains, particularly in uncomplicated appendicitis, with some evidence suggesting no reduction—and possible increase—in infection rates. Similarly, whereas peritoneal lavage is a commonly performed procedure, its effectiveness is disputable, with some studies proving standardized or antiseptic-based techniques to reduce bacterial load and others advocating plain aspiration alone.

Postoperative care of IAAs remains a key part of managing patients, especially after complicated appendicitis. While small, well-contained abscesses can often be treated with antibiotics alone, larger or symptomatic abscesses usually need percutaneous drainage. Laparoscopic preoperative intervention, when carefully chosen, has proven effective and safe, offering the benefit of minimally invasive techniques. However, high-quality evidence specifically regarding the management of post-appendectomy abscesses is limited, highlighting the need for further research and the creation of standardized treatment guidelines.

Finally, ERAS protocols are becoming more commonly applied in appendectomy, and their evidence base to reduce post-operative pain, shorten hospital stay, and also decrease infectious complications is present. Factors such as early mobilization, minimized drain use, maximized fluid management, and early enteral nutrition appear safe and effective in uncomplicated as well as complicated appendicitis.

In conclusion, the prevention of post-appendectomies infections requires a multifaceted approach, including early diagnosis, tailored surgical techniques, evidence-based utilization of adjuncts, and adherence to ERAS principles. While several practices remain debated, ongoing research continues to refine strategies aimed at improving outcomes in this typical yet potentially complex surgical scenario.

## 8. Conclusions

This review summarizes the current knowledge on various factors that contribute to post-operative infection after appendectomy in adults. By examining the existing literature and international guidelines, it becomes clear how each pre-, intra-, and post-operative factor significantly influences patient outcomes, and that sometimes the surgeon’s preferences are not always based on solid evidence. Clearly, exploring every possible strategy to reduce post-operative infections is of paramount importance.

## Figures and Tables

**Table 1:** Timing of surgical intervention in acute appendicitis and effect on post-operative SSI.

Study	Population	Main Findings	Effect on Post-Operative SSI
Tang et al. (2024) Meta-analysis [8]	Adults > 500,000	Delays >24 h increased complicated appendicitis and general morbidity	No association with SSI
Calpin et al. (2024) Meta-analysis [9]	Adults 232,678	Surgery within 24 h of hospital admission was associated with lower rates of complicated appendicitis, shorter hospital stays, and reduced mortality.	No significant reduction in SSI compared to later surgery

Overall, the above results support a risk-adjusted stratified approach.

**Table 2:** Appendiceal stump closure: technique comparison.

Study	Study Design	Technique Compared	Main Findings	Conclusions
Soll et al. (2016) [12]	Retrospective	Hem-o-lok vs. Endoloop	Lower rate of IAA’S with H-O-L	First large-scale evidence favoring polymeric clips
Vuille et al. (2020) [13]	Retrospective	Hem-o-lok vs. Endoloop vs. Stapler	Similar overall IAA’s rates, but data consistently favored H-O-L	No significant difference clip vs. stapler
Ihnát et al. (2021) [15]	RCT	Hem-o-lok vs. Endoloop vs. Stapler	H-O-L shortest operative time (~38 min); no increase in infection rate	Efficiency benefit with H-O-L
WSES (2020) [7]	International Guidelines [QoE: Moderate, 1B]	Endoloop vs. Stapler vs. Clips	No proven advantage of stapler over endoloop; clips viable and economical in selected cases	Clips suitable for appendix base ≤10 mm

**Table 3:** Peritoneal irrigation: techniques and efficacy.

Study	Study Design	Intervention	Key Finding	Conclusions
Gemici et al., (2020) [21]	RCT	Saline lavage (500 mL) vs. aspiration alone	No significant difference in IAA, wound infection, or hospital stay	Lavage provides no added benefit over aspiration alone
Burini et al., (2021) [22]	Meta-analysis	Suction alone vs. lavage (any type)	Suction alone equivalent for IAA prevention; better operative time, fewer reops	Lavage not superior; suction-only may be preferable
Wang et al., (2021) [23]	Retrospective	Lavage/suction before vs. after appendix extraction	Lower SSI in Group lavage before extraction	Timing of lavage may affect infection risk
Sellappan et al., (2024) PLaSSo trial [24]	RCT	Super-oxidized solution vs. saline for peritoneal	Significant reduction in deep and superficial SSIs with antiseptic solution	Antiseptic lavage is safe and superior to saline in open surgery
Singal et al., (2016) [25]	RCT	Super-oxidized solution vs. saline	Reduced bacterial load and shorter hospital stay with antiseptic solution	Antiseptics may benefit broader peritonitis management

## Data Availability

Data sharing is not applicable to this article as no datasets were generated or analyzed during the current study.

## Abbreviations

The following abbreviations are used in this manuscript:

LA	Laparoscopic appendectomy
SSI	Surgical Site Infection
IAA	Intra-Abdominal Abscess
BMI	Body Mass Index
ICS	Intraoperative Culture Swab
H-O-L	Hem-o-lock
VAT	Visceral Adipose Tissue
ERAS	Enhanced Recovery After Surgery
QoE	Quality of Evidence

## References

[1] Golz R.A., Flum D.R., Sanchez S.E., Liu X., Donovan C., Drake F.T. (2020). Geographic Association Between Incidence of Acute Appendicitis and Socioeconomic Status. JAMA Surg..

[2] Xiao Y., Shi G., Zhang J., Cao J.-G., Liu L.-J., Chen T.-H., Li Z.-Z., Wang H., Zhang H., Lin Z.-F. (2015). Surgical site infection after laparoscopic and open appendectomy: A multicenter large consecutive cohort study. Surg. Endosc..

[3] Giesen L.J.X., Van Den Boom A.L., Van Rossem C.C., Den Hoed P.T., Wijnhoven B.P.L. (2017). Retrospective Multicenter Study on Risk Factors for Surgical Site Infections after Appendectomy for Acute Appendicitis. Dig. Surg..

[4] Cironi K., Albuck A.L., McLafferty B., Mortemore A.K., McCarthy C., Hussein M., Issa P.P., Metz T., Herrera M., Toraih E. (2024). Risk Factors for Postoperative Infections Following Appendectomy of Complicated Appendicitis: A Meta-analysis and Retrospective Single-institutional Study. Surg. Laparosc. Endosc. Percutan. Tech..

[5] (2025). NHSN Patient Safety Component Manual. https://www.cdc.gov/nhsn/pdfs/pscmanual/pcsmanual_current.pdf.

[6] Walędziak M., Lasek A., Wysocki M., Su M., Bobowicz M., Myśliwiec P., Astapczyk K., Burdzel M., Chruściel K., Cygan R. (2019). Risk factors for serious morbidity, prolonged length of stay and hospital readmission after laparoscopic appendectomy—Results from Pol-LA (Polish Laparoscopic Appendectomy) multicenter large cohort study. Sci. Rep..

[7] Di Saverio S., Podda M., De Simone B., Ceresoli M., Augustin G., Gori A., Boermeester M., Sartelli M., Coccolini F., Tarasconi A. (2020). Diagnosis and treatment of acute appendicitis: 2020 update of the WSES Jerusalem guidelines. World J. Emerg. Surg..

[8] Tang G., Zhang L., Xia L., Zhang J., Chen R., Zhou R. (2025). Preoperative in-hospital delay increases postoperative morbidity and mortality in patients with acute appendicitis: A meta-analysis. Int. J. Surg..

[9] Calpin G.G., Hembrecht S., Giblin K., Hehir C., Dowling G.P., Hill A.D.K. (2024). The impact of timing on outcomes in appendicectomy: A systematic review and network meta-analysis. World J. Emerg. Surg..

[10] Ninh A., Wood K., Bui A.H., Leitman I.M. (2019). Risk Factors and Outcomes for Sepsis after Appendectomy in Adults. Surg. Infect..

[11] Peng H., Zhang J., Cai C., Fang X., Wu J. (2018). The Influence of Carbon Dioxide Pneumoperitoneum on Systemic Inflammatory Response Syndrome and Bacterial Translocation in Patients With Bacterial Peritonitis Caused by Acute Appendicitis. Surg. Innov..

[12] Soll C., Wyss P., Gelpke H., Raptis D.A., Breitenstein S. (2016). Appendiceal stump closure using polymeric clips reduces intra-abdominal abscesses. Langenbecks Arch. Surg..

[13] Vuille-dit-Bille R., Soll C., Mazel P., Staerkle R.F., Breitenstein S. (2020). Appendiceal stump closure with polymeric clips is a reliable alternative to endostaplers. J. Int. Med. Res..

[14] Guldogan C.E., Sarp G., Soyer Guldogan E. (2024). Comparison of Hem-o-lok and Endoloop for Appendiceal Stump Closure in Laparoscopic Appendectomy: An Observational Retrospective Study. Ann. Ital. Chir..

[15] Ihnát P., Tesař M., Tulinský L., Ihnát Rudinská L., Okantey O., Durdík Š. (2021). A randomized clinical trial of technical modifications of appendix stump closure during laparoscopic appendectomy for uncomplicated acute appendicitis. BMC Surg..

[16] Hue C.S., Kim J.S., Kim K.H., Nam S.-H., Kim K.W. (2013). The usefulness and safety of Hem-o-lok clips for the closure of appendicular stump during laparoscopic appendectomy. J. Korean Surg. Soc..

[17] Tang Y., Liu J., Bai G., Cheng N., Deng Y., Cheng Y. (2025). Abdominal drainage to prevent intraperitoneal abscess after appendectomy for complicated appendicitis. Cochrane Database Syst. Rev..

[18] Akingboye A.A., Davies B., Tien T. (2019). Pus Samples in Complicated Appendicitis: An Important Investigation or a Waste of Resources: A Prospective Cohort Study. Scand. J. Surg..

[19] Peña M.E., Sadava E.E., Laxague F., Schlottmann F. (2020). Usefulness of intraoperative culture swabs in laparoscopic appendectomy for complicated appendicitis. Langenbecks Arch. Surg..

[20] Quintero-Riaza V.M., Chancí-Drago R., Guzmán-Arango N., Posada-Moreno P., López-Sandoval T., Ramírez-Sánchez I.C., Vanegas-Munera J.M. (2023). Aerobic Intraoperative Abdominal Cavity Culture Modifies Antibiotic Therapy and Reduces the Risk of Surgical Site Infection in Complicated Appendicitis with Peritonitis. J. Gastrointest. Surg..

[21] Gemici E., Bozkurt M.A., Sürek A., Seyhun C., Güneş M.E. (2020). Laparoscopic Lavage Versus Aspiration Alone in Perforated Acute Appendicitis: A Prospective Randomized Controlled Study. Surg. Laparosc. Endosc. Percutan. Tech..

[22] Burini G., Cianci M.C., Coccetta M., Spizzirri A., Di Saverio S., Coletta R., Sapienza P., Mingoli A., Cirocchi R., Morabito A. (2021). Aspiration versus peritoneal lavage in appendicitis: A meta-analysis. World J. Emerg. Surg..

[23] Wang B., Shi L., Fu W., Liu T. (2021). Effects of Sequence of Irrigation, Suction, and Extraction in Cases of Acute Purulent Appendicitis or Gangrenous Perforated Appendicitis After Laparoscopic Appendectomy. J. Laparoendosc. Adv. Surg. Tech..

[24] Sellappan H., Alagoo D., Loo C., Vijian K., Sibin R., Chuah J.A. (2024). Effect of peritoneal and wound lavage with super-oxidized solution on surgical-site infection after open appendicectomy in perforated appendicitis (PLaSSo): Randomized clinical trial. BJS Open.

[25] Singal R., Dhar S., Zaman M., Singh B., Singh V., Sethi S. (2016). Comparative Evaluation of Intra-Operative Peritoneal Lavage with Super Oxidized Solution and Normal Saline in Peritonitis Cases; Randomized Controlled Trial. Maedica.

[26] Turner S.A., Jung H.S., Scarborough J.E. (2019). Utilization of a specimen retrieval bag during laparoscopic appendectomy for both uncomplicated and complicated appendicitis is not associated with a decrease in postoperative surgical site infection rates. Surgery.

[27] Mendoza-Sagaon M., Hamitaga F., Hurni Y., Voumard N. (2016). Appendix extraction after laparoscopic appendectomy in children: An easy, safe, and inexpensive technique. J. Pediatr. Surg..

[28] Sartelli M., Coccolini F., Kluger Y., Agastra E., Abu-Zidan F.M., Abbas A.E.S., Ansaloni L., Adesunkanmi A.K., Atanasov B., Augustin G. (2021). WSES/GAIS/SIS-E/WSIS/AAST global clinical pathways for patients with intra-abdominal infections. World J. Emerg. Surg..

[29] Cho J., Park I., Lee D., Sung K., Baek J., Lee J. (2016). Antimicrobial treatment after laparoscopic appendectomy for preventing a post-operative intraabdominal abscess: A Prospective Cohort Study of 1817 patients. Int. J. Surg..

[30] Sawyer R.G., Claridge J.A., Nathens A.B., Rotstein O.D., Duane T.M., Evans H.L., Cook C.H., O’Neill P.J., Mazuski J.E., Askari R. (2015). Trial of Short-Course Antimicrobial Therapy for Intraabdominal Infection. N. Engl. J. Med..

[31] Kassi F., Dohan A., Soyer P., Vicaut E., Boudiaf M., Valleur P., Pocard M. (2014). Predictive factors for failure of percutaneous drainage of postoperative abscess after abdominal surgery. Am. J. Surg..

[32] Allaway M.G.R., Clement K., Eslick G.D., Cox M.R. (2019). Early Laparoscopic Washout may Resolve Persistent Intra-abdominal Infection Post-appendicectomy. World J. Surg..

[33] Casas M.A., Laxague F., Schlottmann F., Sadava E.E. (2021). Re-laparoscopy for the treatment of complications after laparoscopic appendectomy: Is it possible to maintain the minimally invasive approach?. Updat. Surg..

[34] Nechay T., Sazhin A., Titkova S., Tyagunov A., Anurov M., Melnikov-Makarchuk K., Tyagunov A. (2020). Evaluation of enhanced recovery after surgery program components implemented in laparoscopic appendectomy: Prospective randomized clinical study. Sci. Rep..

[35] Li Z.-L., Ma H.-C., Yang Y., Chen J.-J., Wang Z.-J. (2024). Clinical study of enhanced recovery after surgery in laparoscopic appendectomy for acute appendicitis. World J. Gastrointest. Surg..

[36] Wu Z., Ge X., Shi D. (2024). ERAS and Gastrointestinal Site Infections: Insights from a Comprehensive Systematic Review and Meta-Analysis. Surg. Infect..

